# Prevalence, risk factors and psychological consequences of workplace violence among health workers in the Greater Accra region, Ghana: a cross-sectional study

**DOI:** 10.1186/s12889-024-17962-8

**Published:** 2024-02-22

**Authors:** Philip Apraku Tawiah, Emmanuel Appiah-Brempong, Paul Okyere, Geoffrey Adu-Fosu, Mary Eyram Ashinyo

**Affiliations:** 1 Department of Occupational and Environmental Health & Safety, School of Public Health, College of Health Sciences, Kwame Nkrumah University of Science and Technology, Kumasi, Ghana; 2 Department of Pharmacognosy and Herbal Medicine, School of Pharmacy, University of Health and Allied Sciences, Ho, Ghana; 3 Department of Health Promotion & Disability Studies, School of Public Health, College of Health Sciences, Kwame Nkrumah University of Science and Technology, Kumasi, Ghana; 4 Physiotherapy Unit, Diagnostic and Rehabilitation Directorate, Ho Teaching Hospital, Ho, Ghana; 5 Department of Quality Assurance– Institutional Care Division, Ghana Health Service Headquarters, Accra, Ghana; 6 Department of Maternal and Child Health, Gilling’s School of Global Public Health, University of North Carolina, Chapel Hill, NC USA

**Keywords:** Verbal abuse, Health worker safety, Workplace safety, West Africa, On-call duties

## Abstract

**Background:**

Globally, close to one-third of all workplace violence (WV) occurs in the health sector. Exposure to WV among healthcare professionals in Ghana has been widely speculated, but there is limited evidence on the problem. This study therefore investigated WV, its risk factors, and the psychological consequences experienced by health workers in Ghana.

**Methods:**

An analytic cross-sectional study was conducted in the Greater Accra region from January 30 to May 31, 2023, involving selected health facilities. The participants for the study were selected using a simple random sampling technique based on probability proportional-to-size. The data analyses were performed using STATA 15 software. Logistic regression analyses were employed to identify the factors associated with WV, considering a significance level of *p*-value < 0.05.

**Results:**

The study was conducted among 607 healthcare providers and support personnel across 10 public and private hospitals. The lifetime career, and one-year exposure to any form of WV was 414 (68.2%) [95% CI: (64.3-71.9%)] and 363 (59.8%) [95% CI: (55.8-63.7%)], respectively. Compared to other forms of WV, the majority of healthcare workers, 324 (53.4%) experienced verbal abuse within the past year, and a greater proportion, 85 (26.2%) became ‘super alert’ or vigilant and watchful following incidents of verbal abuse. Factors significantly linked to experiencing any form of WV in the previous 12 months were identified as follows: older age [AOR = 1.11 (1.06, 1.17)], working experience [AOR = 0.91 (0.86, 0.96)], having on-call responsibilities [AOR = 1.75 (1.17, 2.61)], and feeling adequately secure within health facility [AOR = 0.45 (0.26, 0.76)].

**Conclusion:**

There was high occurrence of WV, and verbal abuse was the most experienced form of WV. Age, work experience, on-call duties, and security within workplace were associated with exposure to WV. Facility-based interventions are urgently needed to curb the incidence of WV, especially verbal abuse.

**Supplementary Information:**

The online version contains supplementary material available at 10.1186/s12889-024-17962-8.

## Introduction

Workplace violence among healthcare personnel has been reported as one of the most challenging public health threats across both high-income and low-middle-income countries, with more cases of violence occurrence in developing countries due to the execrable conditions of their health sector’s care and services [1, 2]. Also, close to one-third of all workplace violence occurs in the health sector worldwide [3]. Almost 88.0% of health workers in third-world countries are exposed to various forms of workplace violence, including work abuse, bullying, and attacking with objects [4]. According to the Australian Institute of Criminology, the above accounts contribute to the label of the healthcare industry as the most violent in the world [5]. Workplace violence in the health sector sabotages the dignity, safety, health, and social well-being of healthcare providers and supporting staff [6, 7]. Additionally, healthcare facilities suffer from absenteeism, loss of experts, payment of compensation, psychological consequences, and employee turnover intention due to workplace violence [3].

The incidence of workplace violence has risen to an endemic level, making almost all categories of healthcare professionals susceptible; however, nurses are more exposed [8, 9]. The global prevalence of any form of workplace violence among healthcare workers stood at 61.9%, per the findings of a systematic review and meta-analysis published in 2019 [10]. Also, the prevalence of verbal abuse, threats, physical abuse, and sexual harassment was reported as 57.6%, 33.2%, 24.4%, and 12.4%, respectively [10]. In Africa, varied but high prevalence of workplace violence, ranging from 9 to 100% has been reported in various studies with the highest exposure to workplace violence in South Africa and Egypt [11], reporting within the range of 54.0-100.0% and 59.7–86.1%, respectively. The majority of perpetrators of workplace violence are identified as patients, relatives of patients, co-workers, and supervisors [11].

Socio-demographic, occupational, and organizational factors can be linked to workplace violence among health workers through complex interactions. Socio-demographic factors such as age or gender may influence an individual’s vulnerability to workplace violence, occupational factors such as job demands and workload can contribute to stress and one’s susceptibility to violence incidences, while organizational factors like lack of security measures may create an environment conducive to violence [12, 13]. In a study, gender, age, duty schedule, marital status, and department were risk factors that accounted for the occurrence of workplace place violence among health workers [11]. Understanding these links is crucial for developing effective prevention strategies and support systems for health workers.

In the Ghanaian setting, exposure to workplace violence among health workers has been widely rumoured, but there is insufficient evidence on the issue [14]. A few available studies depicted that about 9.0–73.9% cases of workplace violence are experienced by health workers in Ghana [14–17]. Boafo et al.‘s study revealed that 12.0% and 52.2% of nurses in Ghana are sexually harassed and verbally abused, respectively. Another study in Ghana revealed that 24.4% of health workers in a typical district hospital experienced some form of workplace violence [17]. These few estimates ascertain the existence of workplace violence in the Ghanaian health sector; nonetheless, the empirical evidence is inadequate to inform policy. Insufficient data on workplace violence coupled with underreporting of violence incidences, especially in developing countries such as Ghana, aggravates the problem by concealing the true evidence of exposure to workplace violence.

To the best of our knowledge, only a few studies have investigated the occurrence of workplace violence among workers in the healthcare sector of Ghana [3, 17]. A study extensively studied workplace violence using only nurses as study participants [3]. Another study just estimated exposure to workplace violence without investigating its predisposing factors [17]. Obviously, no baseline study has assessed workplace violence and its predisposing factors among health workers in the Ghanaian industry. Therefore, this study determined the prevalence and risk factors of workplace violence, as well as the psychological consequences experienced by healthcare providers and ancillary staff of hospitals in the densely populated Greater Accra region of Ghana.

## Approach and methodology

### Study design, participants and setting

A facility-based analytic cross-sectional study design, and a quantitative approach was employed for this investigation. A survey was conducted among a variety of healthcare professionals, including doctors, nurses, midwives, medical laboratory staff, physiotherapists, health care assistants, orderlies, and laundry workers. This survey was carried out within ten district and private hospitals located in the Greater Accra region of Ghana. Among these hospitals were the Weija-Gbawe Municipal Hospital, Ashaiman Community Hospital, Pentecost Hospital, Sakumono Community Hospital, Nyaho Medical Centre, Shai-Osudoku Hospital, Tema General Hospital, Achimota Hospital, LEKMA Hospital, and Ga North Municipal Hospital. Of these, six were public hospitals, while the remaining hospitals were private-based facilities. These hospitals are major healthcare facilities in their area of location (district) and offer Out-Patients Department (OPD), ante-natal and family planning, dental services, eye care, laboratory, ear-nose-and throat care, radiology, and dermatology services, as well as surgeries. The bed capacity of these facilities ranges from 50 to 500, whereas the total population of healthcare professionals and housekeeping staff ranges from 77 to 579.

The study region, Greater Accra, is one of the sixteen administrative regions in Ghana [18]. It houses over 1500 health care facilities, including a teaching hospital, regional and district hospitals, polyclinics, health centres, and community-based health planning and services (CHPS) compounds. Again, the region hosts about 30.6% of all healthcare providers (medical officers, midwives, nurses, and pharmacists) in Ghana [19]. The Greater Accra Region is the most populated region in Ghana with an estimated population of 5,455,692 in the year 2021 [20]. Moreover, close to 91.7% of its residents are living in urban areas. Due to in-migration and a high population growth rate, the Greater Accra region is regarded as the region with the highest population density of approximately 1681.3 persons per sq. km [20].

### Sample size determination

The Cochran formulae [21], ${N}_{o}=\frac{{z}^{2}pq}{{d}^{2}}$, for sample size estimation was used to predict the sample size for the study. Using z = constant for the 95% confidence interval given as 1.96, *p* = proportion of the population (52.7%) that experienced the outcome (workplace verbal abuse) of a previous study conducted in Ghana [15], q = (1-p) and d = margin of error, estimated at 5% for this study, sample size, ${N}_{o},$ was estimated to be 383. After applying a design effect of 2.0 [22, 23], finite correction population formula proposed by Neyman [24, 25] and an anticipated 10% non-response rate, we arrived at a final sample size of 673. Nonetheless, six hundred and seven (607) healthcare workers participated in the study. This number reflects a response rate of 90.2%. Insufficient provision of monetary compensation as the main contributing factor to non-achievement of 100% response rate.

### Sampling procedure

A multistage sampling method was the overarching technique employed in this study. The Greater Accra region of Ghana was purposively selected, followed by the random selection of districts, hospitals and study participants. Additionally, probability proportional-to-size guided the selection of districts from region, hospitals from districts, and study participants based on their occupation. Healthcare services are operational in all 29 districts of the Greater Accra region, which is made up of two metropolitan assemblies, twenty-three municipal assemblies, and four ordinary districts. Ten out of the 29 zones, which contribute to more than 30.0% of the total districts were selected. Also, ten out of 17 major hospitals included in the sampling frame were selected through simple random sampling. One major hospital was selected to represent each district for the study; however, in districts where there were two or three major hospitals, simple random sampling was used to select one. The 2021 annual outpatient department (OPD) attendance generated from the District Health Information Management System (DHIMS) [26] guided the selection of major hospitals into the sampling frame. A stratified random sampling method was used to select study participants from their profession.

### Inclusion and exclusion criteria

The study was restricted to healthcare providers and ancillary staff: doctors, nurses, midwives, medical laboratory staff, physiotherapists, health care assistants, laundry workers and orderlies. Additionally, these group of workers should have worked in a hospital for at least twelve months. Any other health worker apart from those stated in the inclusion criteria, such as administrators, radiographers, dieticians, and health students among others were excluded from the study. Also, newly recruited health professionals were not allowed to partake in the study.

### Study questionnaire and data collection

A structured questionnaire was used as the data collection tool for this study, portions of this tool, especially the part relating to psychological effects of physical, verbal and sexual violence on Health Workers, were adapted from the International Labour Office, the International Council of Nurses, the World Health Organization, and Public Services International’s health sector workplace violence questionnaire [27] and the National Institute for Occupational Safety and Health, US Centre for Disease Control and Prevention’s Healthcare workers Safety and Health Survey questionnaire [28]. The questionnaire comprised of closed-ended and open-ended questions, and it was methodically organized into five distinct sections. Section I was dedicated to collecting pertinent socio-demographic and lifestyle characteristics of the respondent. Section II addressed occupational factors, while Section III delved into organisational and intervention factors. Section IV explored workplace violence, and finally, Section V was devoted to investigating the psychological consequences of workplace violence. These sections I, II, III, IV and V comprised of 13, 8, 7, 5, and 9 questions, respectively.

The questionnaire was pretested among sixty healthcare providers and ancillary staff at the Ho Teaching Hospital. Subsequent to the pre-testing phase, the questions were thoroughly reviewed based on the valuable feedback provided by the study respondents and other relevant occupational health and safety stakeholders. The data was collected through the distribution of a self-administered paper questionnaire, which was disseminated to the participants after an initial interaction about the study. Additionally, the participants were duly advised to complete the questionnaire at their earliest convenience. For participants who required assistance in completing the questionnaire, research assistants conducted interviews to facilitate the process. The data was entered into Open Data Kit, an electronic platform [29]. The entire data collection process was conducted between the period of January 30 and May 31, 2023.

### Data management and analysis

The data utilized in this study were exported from the Open Data Kit electronic platform [29] and imported into the STATA SE version 15 (64-bit) statistical analysis software [30] for both cleaning and analysis. A thorough error-check was performed on the data prior to its analysis, followed by the necessary cleaning procedures. In order to confirm the presence or absence of missing values, frequencies were conducted on all variables. Additionally, quantitative variables underwent skewness and kurtosis tests to determine their suitability for either parametric or non-parametric tests.

Frequencies and percentages were employed to provide a summary of categorical variables, whereas continuous variables were summarized using median and interquartile range. The independent variables, which consisted of socio-demographic and lifestyle characteristics, occupational factors, organisational factors, intervention strategies, and psychological consequences of workplace violence, were presented in tabular form. Conversely, the dependent variables, which were lifetime and 12-month prevalence of workplace violence encompassing physical assault, verbal abuse, and sexual harassment, were displayed using bar graphs. Statistical indicators such as crude odds ratio, adjusted odds ratio, 95% confidence intervals, and *p*-values were computed using a two-sided test.

Chi-square, Fisher’s exact, and Mann-Whitney U tests were employed to establish an initial association between the prevalence of workplace violence (occurrence of at least one incident of physical violence, verbal abuse, or sexual harassment within the past year) and independent variables. Bivariate and multiple logistic regression analyses were conducted to confirm the relationship between independent variables and the prevalence of workplace violence. Additionally, variables that demonstrated significance at a *p*-value of less than 0.05 on the tests of association were considered in the multiple logistic regression model.

## Results and interpretation

### Socio-demographic and lifestyle characteristics of health workers

Table 1 provides an overview of socio-demographic and lifestyle characteristics of health workers from ten major hospitals in the Greater Accra region of Ghana. Among the 607 health workers who took part in the study, the largest portions belonged to the healthcare provider category and nursing profession, accounting for 543 (89.3%) and 332 (54.7%) respectively. Approximately 312 (51.4%) of the participants fell into the age range of 30 to 40 years, with a median age of 32 years and an interquartile range of 28 to 37 years. Majority of the participants, 499 (82.2%), were females, and nearly half of them, 300 (49.4%), were married. Most respondents, 558 (91.93%), had achieved tertiary education, while a significant portion, 283 (46.6%), had less than 5 years of work experience. The median work experience was 5 years, with an interquartile range of 3 to 12 years. Also, 532 (87.6%) of the participants were employed in public health facilities, and 512 (84.35%) were permanently hired. Similarly, a substantial portion of the respondents, 493 (81.22%), worked for 5 days or fewer per week. More than a tenth of the participants, 100 (16.47%), were supervisors. Almost two-thirds of study participants, 395 (65.1%), occasionally experienced family conflicts, and majority, 531 (87.5%), were not consumers of alcohol. Most of the study respondents, 206 (34.0%), were earning within 2000–3999 cedi ($168–336), with the median income of 2000 cedi ($168), and an interquartile range of 1000 to 3000 cedi ($84–252).

**Table 1:** Socio-demographic and lifestyle characteristics of health workers

Characteristics	Frequency (607)	Percentage (%)
**Gender**		
Female	499	82.21
Male	108	17.79
**Age**		
Median (IQR)	32.0	28.0–37.0
Younger than 30	211	34.76
30–39	312	51.40
40–49	68	11.20
50 and older	16	2.64
**Occupation**		
Doctor	41	6.75
Nurse	332	54.70
Midwife	130	21.42
Laboratory staff	34	5.60
Physiotherapist	5	0.82
Orderlies	54	8.90
Laundry staff	2	0.33
Healthcare Assistant	9	1.48
**Type of health worker**		
Healthcare provider	542	89.29
Ancillary staff	65	10.71
**Marital status**		
Single	295	48.60
Married	300	49.42
Divorced/separated/widowed	12	1.98
**Highest educational level**		
Primary/secondary	49	8.07
Tertiary	558	91.93
**Income (GH¢)**		
Median (IQR)	2000	1000–3000
Less than 1000	101	16.64
1000–1999	183	30.15
2000–3999	206	33.94
4000 and above	117	19.28
**Type of health facility**		
Private	75	12.36
Public	532	87.64
**Working experience in health facility**		
Median (IQR)	5.0	3.0–12.0
Less than 5	283	46.62
5–10	109	17.96
Above 10 years	215	35.42
**Type of employment**		
Contract	95	15.65
Permanent	512	84.35
**Current position**		
No position	473	77.92
Supervisor	100	16.47
Head of Department	34	5.60
**Frequency of family conflicts**		
Not at all	212	34.93
Occasionally	395	65.07
**Consumption of alcohol**		
No	531	87.48
Yes	76	12.52
**Working days in a typical week**		
5 and below	493	81.22
Above 5	114	18.78

### Occupational related factors

A little over half of the study participants, 310 (51.1%) and 309 (50.9%), worked overtime, and were on a mix of day, evening and nights shifts, respectively. Additionally, the majority of the study respondents, 570 (93.9%), were on full time employment, and a greater portion of them, 375 (61.8%), were placed on on-call duties. Also, pressure from work was occasionally experienced by the majority, 322 (53.1%), of the participants, and most of them, 244 (40.2%), reported extremely demanding work. Many of the participants, 354 (58.3%), experienced moderate amounts of stress. Few participants, 62 (10.2%), worked in multiple facilities (Table 2).

**Table 2:** Occupational related factors

Characteristics	Frequency (607)	Percentage (%)
**Overtime**		
No	297	48.93
Yes	310	51.07
**Type of shift**		
Day only	282	46.46
Evening/swing only	16	2.64
A mix of day, evening and nights	309	50.91
**On call duties**		
No	375	61.78
Yes	232	38.22
**Type of employment**		
Full time	570	93.90
Part time	37	6.10
**Work in multiple facility**		
No	545	89.79
Yes	62	10.21
**Pressure from work**		
Not at all	28	4.61
Occasionally	322	53.05
Frequently	257	42.34
**Demanding work**		
Not at all	13	2.14
A little bit	25	4.12
Moderately	164	27.02
Quite a bit	161	26.52
Extremely	244	40.20
**Stress**		
Almost no stress at all	12	1.98
A moderate amount of stress	354	58.32
A lot of stress	241	39.70

### Organizational and intervention related factors

A greater number of participants, 431 (71.0%), reported the availability of hazard reporting system in their facilities, and about two-thirds of them, 401 (66.1%), were understaffed in their department. Additionally, a little over half, 310 (51.1%), of them confirmed the availability of policy on workplace violence. Majority of the participants, 548 (90.3%) and 453 (74.6%) felt safe while working, and had a feeling of been adequately secured in their facility, respectively. About two-thirds, 390 (64.3%), of participants were trained on reporting violence, and less than half of them, 254 (41.9%) were trained on how to recognize violence incidences (Table 3).

**Table 3:** Organizational and intervention related factors

Characteristics	Frequency (607)	Percentage (%)
**Reporting system for hazards**		
No	55	9.06
Yes	431	71.00
Don’t know	121	19.33
**Understaffed**		
No	206	33.94
Yes	401	66.06
**Felt safe while working**		
No	59	9.72
Yes	548	90.28
**Felt adequately secured in facility**		
No	154	25.37
Yes	453	74.63
**Policy on workplace violence**		
No	251	41.35
Yes	310	51.07
Don’t know	46	7.58
**Trained on report of violence**		
No	217	35.75
Yes	390	64.25
**Trained to recognize violence**		
No	353	58.15
Yes	254	41.85

### Prevalence of exposure to workplace violence among health workers

**Fig. 1:**
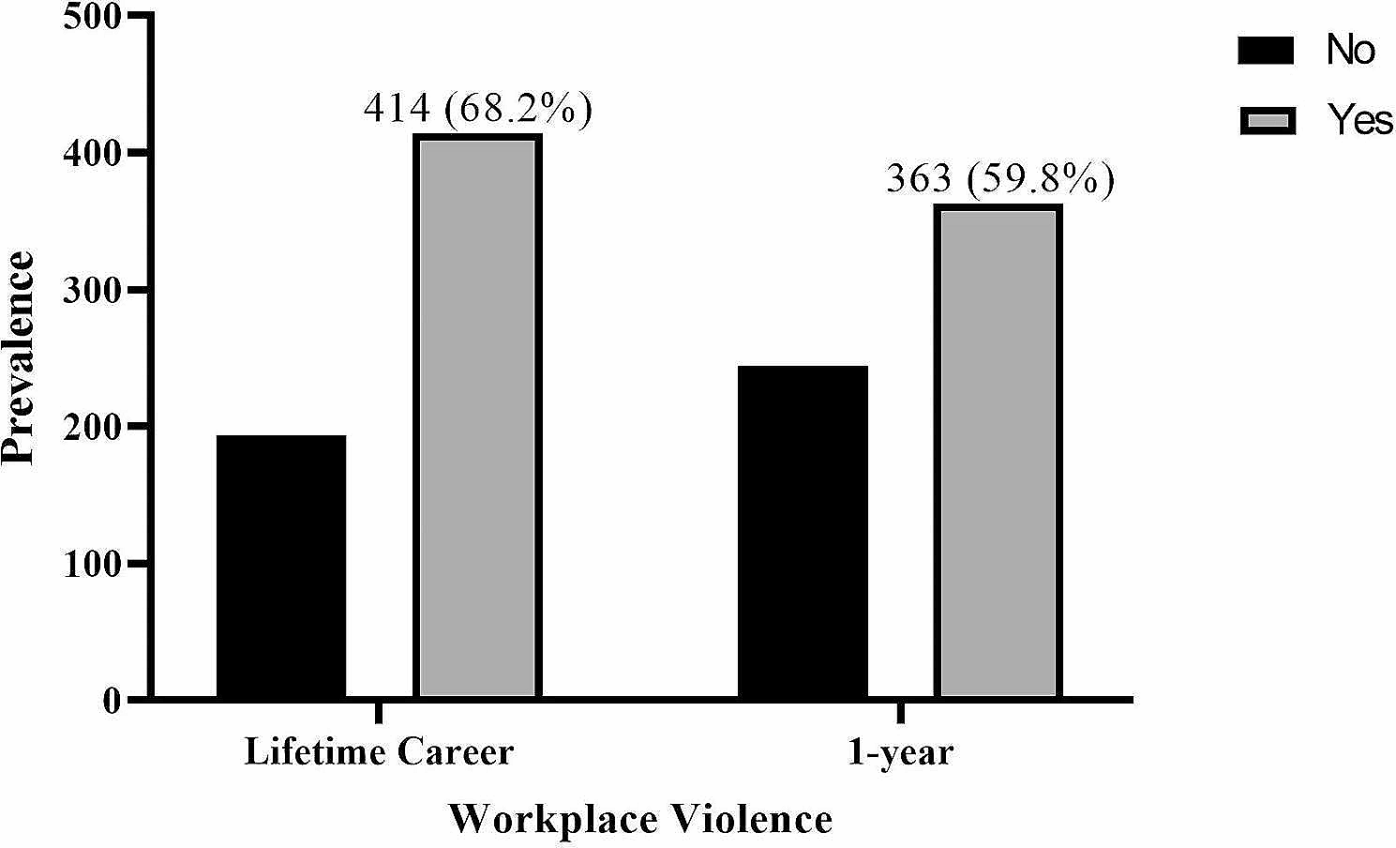
Lifetime career and one-year exposure to workplace violence

**Fig. 2:**
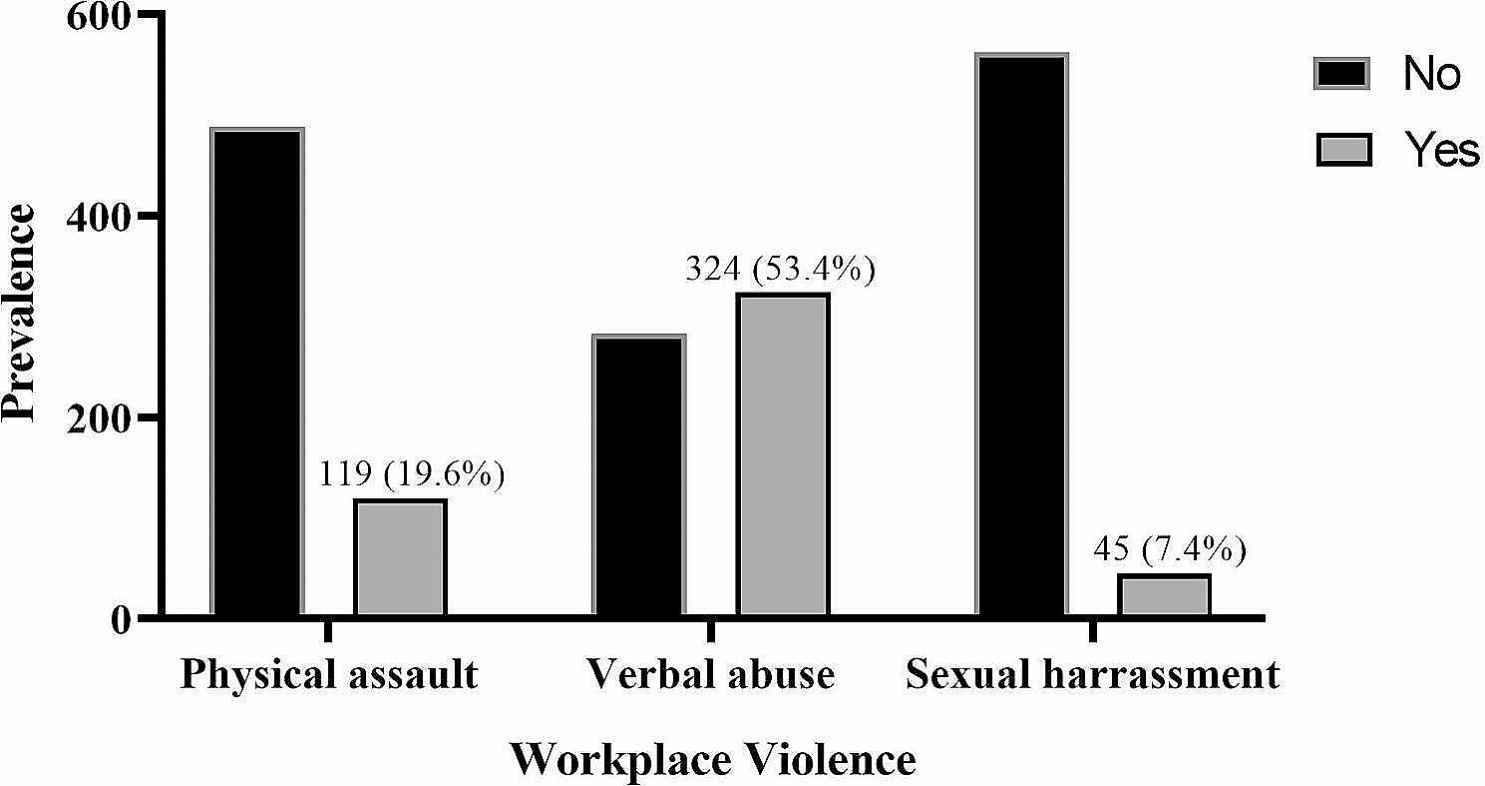
One-year exposure to forms of workplace violence

Majority of the study participants, 414 (68.2%) and 363 (59.8%) were exposed to at least one form of workplace violence in their lifetime career, and the past 1 year, respectively (Fig. 1). Also, the most prevalent workplace violence in the past 12 months was verbal abuse, which was experienced by 324 (53.4%) study participants (Fig. 2).

### Socio-demographic characteristics influencing workplace violence

A significant association was observed between age (t = -5.19, *p*-value = < 0.001), type of health worker (***χ*2** = 10.54, *p*-value = 0.001), highest educational level (***χ*2** = 10.57, *p*-value = 0.001), income (t = -2.83, *p*-value = 0.004), and the occurrence of workplace violence. Also, the relationship between working experience (t = -3.41, *p*-value = < 0.001), type of employment (***χ*2** = 4.03, *p*-value = 0.045), family conflicts (***χ*2** = 5.73, *p*-value = 0.017), consumption of alcohol (***χ*2** = 6.96, *p*-value = 0.008), and the prevalence of workplace violence was significant (Table 4).

**Table 4:** Socio-demographic and lifestyle characteristics influencing workplace violence

Characteristics	N	Workplace violence		χ2/t	*p*-value
		No	Yes		
**Gender**				0.31	0.576
Female	499	198 (39.68)	301 (60.32)		
Male	108	46 (42.59)	62 (57.41)		
**Age**				− 5.19	< 0.001*^b^
Median (IQR)	32.0	30.0 (26.0–35.0)	33.0 (29.0–38.0)		
**Type of health worker**				10.54	0.001*
Healthcare provider	542	230 (42.44)	312 (57.56)		
Ancillary staff	65	14 (21.54)	51 (78.46)		
**Marital status**				2.51	0.298^a^
Single	295	128 (43.39)	167 (56.61)		
Married	300	112 (37.33)	188 (62.67)		
Divorced/separated/widowed	12	4 (33.33)	8 (66.67)		
**Highest educational level**				10.57	0.001*
Primary/secondary	49	9 (18.37)	40 (81.63)		
Tertiary	558	235 (42.11)	323 (57.89)		
**Income (GH¢)**				-2.83	0.004*^b^
Median (IQR)	2000	1500 (1000–3000)	2000 (1500–3500)		
**Type of health facility**				0.08	0.773
Private	75	29 (38.67)	46 (61.33)		
Public	532	215 (40.41)	317 (59.59)		
**Working experience**				-3.41	< 0.001*^b^
Median (IQR)	5.0	4.0 (2.0–10.0)	5.0 (3.0–12.0)		
**Type of employment**				4.03	0.045*
Contract	95	47 (49.47)	48 (50.53)		
Permanent	512	197 (38.48)	315 (61.52)		
**Current position**				1.60	0.450
No position	473	184 (38.90)	289 (61.10)		
Supervisor	100	44 (44.00)	56 (56.00)		
Head of Department	34	16 (47.06)	18 (52.94)		
**Family conflicts**				5.73	0.017*
Not at all	212	99 (46.70)	113 (53.30)		
Occasionally	395	145 (36.71)	250 (63.29)		
**Consumption of alcohol**				6.96	0.008*
No	531	224 (42.18)	307 (57.82)		
Yes	76	20 (26.32)	56 (73.68)		
**Working days in a week**				2.75	0.097
5 and below	493	206 (41.78)	287 (58.22)		
Above 5	114	38 (33.33)	76 (66.67)		

* significant variable (*p*-value < 0.05); ^a^*p*-values calculated from Fishers’ exact test

^b^*p*-values calculated from Mann-Whitney U test; IQR– Interquartile range

### Occupational factors influencing workplace violence

There was a significant association between on-call duties (***χ*2** = 9.68, *p*-value = 0.002), demanding work (***χ*2** = 15.61, *p*-value = 0.003), stress (***χ*2** = 13.27, *p*-value = 0.001), and exposure to workplace violence (Table 5).

**Table 5:** Occupational factors influencing workplace violence

Characteristics	N	Workplace violence		χ2	*p*-value
		No	Yes		
**Overtime**				3.70	0.054
No	297	131 (44.11)	166 (55.89)		
Yes	310	113 (36.45)	197 (63.55)		
**Type of shift**				3.17	0.221^a^
Day only	282	116 (41.13)	166 (58.87)		
Evening/swing only	16	3 (18.75)	13 (81.25)		
A mix of day, evening and nights	309	125 (40.45)	184 (59.55)		
**On call duties**				9.68	0.002*
No	375	169 (45.07)	206 (54.93)		
Yes	232	75 (32.33)	157 (67.67)		
**Type of employment**				0.15	0.697
Full time	570	228 (40.00)	342 (60.00)		
Part time	37	16 (43.24)	21 (56.76)		
**Work in multiple facility**				1.24	0.265
No	545	215 (39.45)	330 (60.55)		
Yes	62	29 (46.77)	33 (53.23)		
**Pressure from work**				5.05	0.080
Not at all	28	13 (46.43)	15 (53.57)		
Occasionally	322	141 (43.79)	181 (56.21)		
Frequently	257	90 (35.02)	167 (64.98)		
**Demanding work**				15.61	0.003*^a^
Not at all	13	1 (7.69)	12 (92.31)		
A little bit	25	13 (52.00)	12 (48.00)		
Moderately	164	80 (48.78)	84 (51.22)		
Quite a bit	161	66 (40.99)	95 (59.01)		
Extremely	244	84 (34.43)	160 (65.57)		
**Stress**				13.27	0.001*
Almost no stress at all	12	7 (58.33)	5 (41.67)		
A moderate amount of stress	354	161 (45.48)	193 (54.52)		
A lot of stress	241	76 (31.54)	165 (68.46)		

* significant variable (*p*-value < 0.05); ^a^*p*-values calculated from Fishers’ exact test

### Organizational factors and intervention strategies influencing workplace violence

Regarding organizational factors, felt safe while working (***χ*2** = 7.37, *p*-value = 0.007), felt adequately secured in facility (***χ*2** = 24.29, *p*-value < 0.001), and policy on workplace violence (***χ*2** = 19.09, *p*-value < 0.001) were significantly associated with exposure to workplace violence. Also, with respect to workplace intervention strategies, trained on violence reporting (***χ*2** = 11.03, *p*-value = 0.001) and training on recognition of violence (***χ*2** = 6.25, *p*-value = 0.012) were significantly related to occurrence of workplace violence (Table 6).

**Table 6:** Organizational factors and intervention strategies influencing workplace violence

Characteristics	N	Workplace violence		χ2	*p*-value
		No	Yes		
**Reporting system for hazards**				1.08	0.582
No	55	24 (43.64)	31 (56.36)		
Yes	431	176 (40.64)	255 (59.16)		
Don’t know	121	44 (36.36)	77 (63.64)		
**Understaffed**				3.83	0.050
No	206	94 (45.63)	112 (54.37)		
Yes	401	150 (37.41)	251 (62.59)		
**Felt safe while working**				7.37	0.007*
No	59	14 (23.73)	45 (76.27)		
Yes	548	230 (41.97)	318 (59.80)		
**Felt adequately secured in facility**				24.29	< 0.001*
No	154	36 (23.38)	118 (76.62)		
Yes	453	208 (45.92)	245 (54.08)		
**Policy on workplace violence**				19.09	< 0.001*
No	251	75 (29.88)	176 (70.12)		
Yes	310	146 (47.10)	164 (52.90)		
Don’t know	46	23 (50.00)	23 (50.00)		
**Trained on report of violence**				11.03	0.001*
No	217	68 (31.34)	149 (68.66)		
Yes	390	176 (45.13)	214 (54.87)		
**Trained to recognize violence**				6.25	0.012*
No	353	127 (35.98)	226 (64.02)		
Yes	254	117 (46.06)	137 (53.94)		

* significant variable (*p*-value < 0.05)

### Factors associated with exposure to workplace violence among study participants

Table 7 provides highlights of the analysis involving predisposing factors and their relationship with the occurrence of workplace violence. In the initial bivariate logistic regression, variables such as age, type of health worker, highest educational level, type of employment, family conflicts, consumption of alcohol, on call duties, demanding work, felt secured like working, felt adequately secured in facility, policy on workplace violence, training on violence reporting, and training on recognition of violence exhibited significant associations with workplace violence. However, upon conducting multivariate logistic regression analysis, age, working experience, on call duties, and felt adequately secured in facility remained linked to the occurrence of workplace violence.

With each additional year of age, there is a 7% rise in the odds of experiencing workplace violence (AOR = 1.11, 95% CI = 1.06–1.17, *p*-value < 0.001), and for a year increase in working experience, there is a 9% reduction in the chances of been exposed to workplace violence (AOR = 0.91, 95% CI = 0.86–0.96, *p*-value < 0.001). Felt secured in facility was associated with lower odds of exposure to workplace violence (AOR = 0.45, 95% CI = 0.26–0.76, *p*-value = 0.003), whilst being responsible for on call duties was not (AOR = 1.75, 95% CI = 1.17–2.61, *p*-value = 0.006).

**Table 7:** Bivariate and multiple logistic regression of risk factors and exposure to workplace violence

Characteristics	Workplace Violence (*n* = 607)				
	N	COR (95% CI)	*p*-value	AOR (95% CI)	*p*-value
**Age**					
Median (IQR)	32.0	1.06 (1.03–1.09)	< 0.001*	1.11 (1.06–1.17)	< 0.001*
**Type of health worker**					
Healthcare provider	542	1		1	
Ancillary staff	65	2.69 (1.45–4.97)	0.002*	2.01 (0.66–6.14)	0.218
**Highest educational level**					
Primary/secondary	49	1		1	
Tertiary	558	0.31 (0.15–0.65)	0.002*	1.04 (0.27–4.04)	0.959
**Income (GH¢)**					
Median (IQR)	2000	1.00 (1.00–1.00)	0.270	1.00 (1.00–1.00)	0.887
**Working experience**					
Median (IQR)	5.0	1.02 (1.00–1.05)	0.066	0.91 (0.86–0.96)	< 0.001*
**Type of employment**					
Contract	95	1		1	
Permanent	512	1.57 (1.01–2.43)	0.046*	1.17 (0.68–2.00)	0.573
**Family conflicts**					
Not at all	212	1		1	
Occasionally	395	1.51 (1.08–2.12)	0.017*	1.43 (0.97–2.11)	0.074
**Consumption of alcohol**					
No	531	1		1	
Yes	76	2.04 (1.19–3.50)	0.009*	1.68 (0.92–3.09)	0.094
**On call duties**					
No	375	1		1	
Yes	232	1.71 (1.22–2.42)	0.002*	1.75 (1.17–2.61)	0.006*
**Demanding work**					
Not at all	13	1		1	
A little bit	25	0.08 (0.01–0.68)	0.021*	0.20 (0.02–1.98)	0.171
Moderately	164	0.09 (0.01–0.69)	0.021*	0.15 (0.02–1.23)	0.077
Quite a bit	161	0.12 (0.02–0.94)	0.044*	0.19 (0.02–1.64)	0.132
Extremely	244	0.16 (0.02–1.24)	0.079	0.13 (0.02–1.08)	0.058
**Stress**					
Almost no stress at all	12	1		1	
A moderate amount of stress	354	1.68 (0.52–5.39)	0.384	1.95 (0.47–8.17)	0.359
A lot of stress	241	3.04 (0.93–9.89)	0.065	3.01 (0.69–13.11)	0.142
**Felt safe while working**					
No	59	1		1	
Yes	548	0.43 (0.23–0.80)	0.008*	0.60 (0.27–1.32)	0.203
**Felt adequately secured in facility**					
No	154	1		1	
Yes	453	0.36 (0.24–0.54)	< 0.001*	0.45 (0.26–0.76)	0.003*
**Policy on workplace violence**					
No	251	1		1	
Yes	310	0.48 (0.34–0.68)	< 0.001*	0.68 (0.41–1.13)	0.139
Don’t know	46	0.43 (0.23–0.81)	0.009*	0.64 (0.29–1.23)	0.162
**Trained on report of violence**					
No	217	1		1	
Yes	390	0.55 (0.39–0.79)	0.001*	0.99 (0.61–1.62)	0.981
**Trained to recognize violence**					
No	353	1		1	
Yes	254	0.66 (0.47–0.91)	0.013*	0.82 (0.53–1.26)	0.354

* significant variable (*p*-value < 0.05); COR- Crude Odds Ratio; AOR– Adjusted Odds Ratio

### Effects of physical, verbal and sexual violence on health workers

The Table 8 illustrates the various psychological effects experienced by health workers after their exposure to different forms of violence. A significant number of study participants, 26 (21.9%) and 85 (26.2%) were extremely ‘super alert’ or watchful and on guard after their experience of physical assault, and verbal abuse, respectively.

**Table 8:** Effects of Physical, Verbal and Sexual Violence on Health Workers

Variable	Physical n (%)	Verbal n (%)	Sexual n (%)
**Repeated disturbing memories of the incident**			
Not at all	54 (45.38)	160 (49.38)	19 (42.22)
A little bit	47 (39.50)	92 (28.40)	16 (35.56)
Moderately	13 (10.92)	38 (11.73)	4 (8.89)
Quite a bit	4 (3.36)	25 (7.72)	6 (13.33)
Extremely	1 (0.84)	9 (2.78)	-
**Avoiding thinking or talking about the incident**			
Not at all	45 (37.82)	105 (32.41)	16 (35.56)
A little bit	46 (38.66)	110 (33.95)	11 (24.44)
Moderately	21 (17.65)	51 (15.74)	7 (15.56)
Quite a bit	5 (4.20)	31 (9.57)	7 (15.56)
Extremely	2 (1.68)	27 (8.33)	4 (8.89)
**Being ‘super alert’ or watchful and on guard**			
Not at all	28 (23.53)	49 (15.12)	16 (35.56)
A little bit	31 (26.05)	62 (19.14)	11 (24.44)
Moderately	18 (15.13)	74 (22.84)	7 (15.56)
Quite a bit	16 (13.45)	54 (16.67)	7 (15.56)
Extremely	26 (21.85)	85 (26.23)	4 (8.89)

## Discussion

This study investigated the prevalence of workplace violence and its associated risk factors, as well as the psychological consequences experienced by health professionals. The prevalence of exposure to at least one form of exposure in the past one year was 59.8%. Verbal abuse was the most (53.4%) experienced workplace violence. Being older, and responsible for on call duties were associated with higher odds of exposure to workplace violence, whilst higher work experience, and felt adequately secured in a healthcare facility were associated with lower odds of exposure to workplace violence. Also, a substantial number of health workers were extremely ‘super alert’ or watchful and on guard following their exposure to physical assault and verbal abuse.

In this current study, 59.8% of health workers were exposed to at least one kind of violence in their work settings. The finding was consistent with a study conducted in mainland China (56.4%) [31], and a systematic review among healthcare professionals in Africa (59.2%) [10]. Nonetheless, our finding was lower than studies carried out in Jordan (65.5%) [32], Chile (71.3%) [33], Gambia (62.1%) [34], Malawi (71.0%) [35], and Congo (80.1%) [36]. Also, our outcome was higher than studies conducted in Saudi Araba (47.8%) [37], Botswana (44.1%) [38] and Ghana (24.4%) [17]. The consistently high prevalence of workplace violence may be due to increase workload, work-related stressors, staff shortages, exposure to violent individuals, mental health challenges, and lack of strong violence prevention [39–41]. However, the variations in findings may be due to differences in study settings, workplace violence assessment tools, cultural differences and nature of healthcare system.

Additionally, per the findings of this study, verbal abuse was the most experienced form of workplace violence. Numerous studies [3, 10, 15, 32, 37, 42] conducted across the globe confirm this outcome of the study. A lot of factors can contribute to this observation. Some of these may include highly stressful environment emanating from long working hours, high patient loads, critical decision and emotional charged situations at work environment [43, 44]. Breakdown in communication between health professionals, and patients and their families may also be a factor contributing to higher occurrence of verbal abuse [45]. Also, lack of resources such as shortage of staff, and waiting times may contribute to tension and lead to likelihood of verbal abuse [46, 47]. Further, hierarchical structures may lead to power imbalance and maltreatment, which may comprise verbal abuse [48, 49].

Also, it was found in our study that older healthcare workers had a greater odd of experiencing workplace violence. Our result was similar to a study conducted in Ethiopia [50]. However, most studies conducted in other parts of the world including China [10], Egypt [51] and Jordan [32] reported findings opposite to ours. These variations in results may be due to different study participants. Some studies may not have included healthcare ancillary staff, who are likely to be older people. In the Ghanaian setting, majority of the healthcare ancillary staff are older people above 40 years, and they are likely to experience workplace violence from their colleagues or patients and their relatives compared to the other health professionals because they are not accorded much respect in a healthcare facility. On the other hand, younger professionals might encounter challenges in handling tough scenarios due to their limited experience, or they could be considered as having less authority by patients or their relatives, and may expose them to violence at the workplace.

Further, this current study found that highly experienced healthcare professionals were associated with lower odds of exposure to workplace violence. This finding was coherent with studies conducted among Ethiopian [52] and Italian health workers [53]. Nevertheless, it was different from a study carried out among Chinese healthcare personnel [54]. Health workers with varying working experience are at risk of exposure, but generally speaking, experienced health workers are often less exposed to workplace violence due to their well-developed skills in de-escalating tense situations, ability to anticipate and manage potential conflicts, and familiarity with the protocols and procedures that help maintain a safe and respectful environment [55].

Furthermore, in this study, on-call responsibility was associated with higher exposure to workplace violence. This result supports some studies conducted in Southwest China [56] and India [57]. It’s crucial to emphasize that the likelihood of encountering workplace violence can differ based on the particular situation and job setting. Healthcare professionals who are available for duty at any time might encounter distinct obstacles and possible hazards, like managing upset or inebriated patients during overnight hours [58].

Moreover, according to our study, ‘felt secured’ in one’s facility was associated with lower odds of exposure to workplace violence. A couple of studies conducted in different parts of the world have confirmed this finding of our study [59–62]. Ensuring a sense of safety within a healthcare facility is of utmost importance for professionals given the potential risks of workplace violence. This assurance improves professionals’ ability to concentrate on patient care, lessens stress levels, strengthens staff retention, and cultivates a positive organizational atmosphere [63, 64]. Also, the provision of a secure setting empowers professionals to effectively manage difficult circumstances, provides valuable support resources, and reduces potential hazards.

Finally, this current study found that a considerable number of healthcare workers were extremely ‘super alert’ and on guard following their exposure to physical and verbal violence. This finding was similar to a study conducted in Ghana among nurses [15], the consistency of study findings may be due to the similarity of study settings. Health workers are often alert and on guard after exposure to physical and verbal violence due to the potential for ongoing threats and the psychological impact of such experiences. Acts of physical violence and verbal abuse can leave healthcare professionals feeling disturbed, and may create a sense of unending threat, leading to a need for heightened awareness and vigilance [65]. Additionally, the unpredictable nature of such incidents and the potential for recurring violence contribute to the need for health workers to remain alert and prepared to respond to any future threats [65]. The ongoing psychological burden of verbal violence, as highlighted in a study on the experiences of healthcare workers, also underscores the need for active implementation of effective strategies and policies at the institutional level to address and reduce the impact of such violence [66].

There were some few limitations to the study. The adoption of a cross-sectional study methodology implies that it is incapable of establishing conclusive cause-and-effect relationships or determining the order of causality among varying factors. Additionally, the investigation is vulnerable to recall bias, given that respondents were queried regarding occurrences that transpired in the preceding 12 months. Generalization of the study finding may be limited to major hospitals.

## Conclusion

The frequency of healthcare providers and ancillary staff experiencing at least one form of exposure in the past year was reported as being elevated. Notably, verbal abuse was the most prevalent form of workplace violence. Furthermore, advanced age and on-call responsibilities were associated with greater odds of exposure to workplace violence, whereas advanced work experience, and perceived facility security were associated with lower odds of workplace violence exposure. Additionally, a considerable number of individuals exhibited a heightened state of vigilance and caution subsequent to experiencing physical assault and verbal abuse. Facility managers should consider employing more health workers to reduce the number of workers for on-call duties. The study also recommends the strengthening of the existing workplace violence policies to target ways of curbing the incidence of verbal abuse in our healthcare facilities. Finally, future studies should focus on rigorous study designs to confirm the findings of this study.

### Electronic supplementary material

Below is the link to the electronic supplementary material.

Supplementary Material 1

## Data Availability

The datasets analysed during the current study are available from the corresponding author on reasonable request.

## Abbreviations

PPE: Personal Protective Equipment
WPV: Workplace Violence
