# Supplementary material for: Prevalence, risk factors and psychological consequences of workplace violence among health workers in the Greater Accra region, Ghana: a cross-sectional study
Source: BMC Public Health. 2024 Feb 22;24:563. doi: 10.1186/s12889-024-17962-8 (PMC10882733; doi:10.1186/s12889-024-17962-8)
Supplement: Supplementary file 1 — Supplementary Material 1 [file 12889_2024_17962_MOESM1_ESM.pdf]

STUDY QUESTIONNAIRE

Questionnaire **Serial no.: ……….**

Topic: Exposure to Selected Occupational Health Hazards among Healthcare Providers and Ancillary Staff in the Greater Accra Region of Ghana

Introduction: My name is Philip Apraku Tawiah, a PhD student of the School of Public Health, KNUST, Kumasi. I am conducting a study on the topic "Exposure to Selected Occupational Health Hazards among Healthcare Providers and Ancillary Staff in the Greater Accra region of Ghana". This study is strictly for academic purposes and funded by the student. The purpose of this research is to investigate the exposure to needlestick injuries, splash of body fluids, workplace violence and lower back pain. I kindly ask for your assistance in completing this study questionnaire. For any concern, please feel free to contact me on 0242164519 or the chairman, committee on human research and publication ethics, Kumasi. Tel: 0205453785; 0322063248.

Consent: I have read the information on this study / research or have had it translated into a language I understand. I have also talked it over with the interviewer to my satisfaction. I understand that my participation is voluntary (not compulsory) and any information provided will be used only for this purpose. I also understand that the information will be treated with high level of confidentiality. I understand that I may freely stop being part of this study at any time without having to explain myself. Kindly select the option 'Yes' to consent to participate in this study.

1. Yes [ ]
2. No [ ]

SECTION I : SOCIO-DEMOGRAPHIC CHARACTERISTICS

No.	Question	Coding category	Skip
001	Which gender describes you?	1. Female<br>2. Male	
002	What is your current age (years) as at last birthday?	……………	
003	Choose your marital status.	1. Single<br>2. Married<br>3. Divorced<br>4. Separated<br>5. Co-habiting<br>6. Widow/widower	
004	What is your current religion?	1. Christianity<br>2. Islamic<br>3. Traditional	

		4. Others (specify)…………….	
005	What is your highest educational level?	1. No formal education<br>2. Primary education<br>3. Secondary education<br>4. Tertiary education	
006	Which option describes your category of worker?	1. Doctor<br>2. Nurse<br>3. Midwife<br>4. Laboratory staff<br>5. Physiotherapist<br>6. Orderlies<br>7. Waste handler<br>8. Laundry staff<br>9. Mortuary staff<br>10. Healthcare assistant<br>11. Others (Specify)…………	
007	Which current unit are you working in?	1. Cleaning and laundry<br>2. Consulting room<br>3. Emergency room<br>4. Inpatient ward<br>5. Intensive care unit<br>6. Laboratory<br>7. Outpatient area<br>8. Theatre<br>9. Mortuary<br>10. Physiotherapy unit<br>11. Others (specify)………	
008	What is the name of facility you work in?	1. Achimota Hospital<br>2. Ashaiman Community Hospital<br>3. Ga North Municipal Hospital<br>4. LEKMA Hospital<br>5. Nyaho Medical Centre<br>6. Pentecost Hospital<br>7. Sakumono Community Hospital<br>8. Shai-Osudoku Hospital<br>9. Tema General Hospital<br>10. Weija-Gbawe Municipal Hospital	
009	How long (in years) have you worked in this facility?	……………………….	
010	Which district does your facility resides in?	1. Ashaiman<br>2. Ga North<br>3. Korle-Klottey<br>4. La-Nkwantanang-Madina<br>5. Ledzokuku<br>6. Okaikwei North<br>7. Shai-Osudoku<br>8. Tema Metropolitan Assembly<br>9. Tema West<br>10. Weija-Gbawe	
011	On average, how much (in Cedi) do you spend in a month?	……………………..	
012	Which type of health care facility do you work in?	1. Private<br>2. Public	

013	How many years have you worked in your entire career?	………………………	
014	What is your type of employment?	1. Contract<br>2. Permanent	
015	What is your current position?	1. No position<br>2. Supervisor<br>3. Head of department<br>4. Others (specify)………	
016	How often do you experience family conflict?	1. Not at all<br>2. Occasionally<br>5. Frequently	

SECTION II – LIFESTYLE CHARACTERISTICS

017	Do you consume alcohol?	1. No<br>2. Yes	
018	Do you exercise frequently?	1. No<br>2. Yes	
019	Averagely, estimate your daily hours of sleep.	…………………………	
020	How many days do you work in a typical week?	…………………………	
021	Do you smoke?	1. No<br>2. Yes	

SECTION III : OCCUPATIONAL FACTORS

No.	Question	Coding category	Skip
022	Do you work overtime?	1. No<br>2. Yes	
023	Which of the following describe your shifts?	1. Day only<br>2. Evening/swing only<br>3. Nights only<br>4. A mix of day, evening and nights	
024	Does your job include 'on call' duties?	1. No<br>2. Yes	
025	In this main work at the facility, do you work:	1. Full-time<br>2. Part-time	
026	Do you work in more than one facility?	1. No<br>2. Yes	
027	How often do you experience pressure from your work?	1. Not at all<br>2. Occasionally<br>3. Frequently	
028	To what extend do you rate the demanding nature of your job?	1. Not at all<br>2. A little bit<br>3. Moderately<br>4. Quite a bit<br>5. Extremely	

029	How much stress have you experienced at work during the past 7 calendar days?	1. Almost no stress at all<br>2. A moderate amount of stress<br>3. A lot of stress	

SECTION IV : ORGANIZATIONAL FACTORS

No.	Question	Coding category	Skip
030	Is a policy on occupational health and safety available in your facility?	1. No<br>2. Yes<br>3. Don't know	
031	Does your facility have a reporting system for exposure to occupational hazards?	1. No<br>2. Yes<br>3. Don't know	
032	Do you have access to needed personal protection equipment?	1. Never<br>2. Rarely<br>3. Sometimes<br>4. Most of the time<br>5. Always	
033	Is there an area designated to dispose medical waste available in your facility?	1. No<br>2. Yes	
034	Are dustbins accessible in your facility?	1. No<br>2. Yes	
035	Are there safety guidelines hanged on walls of facility?	1. No<br>2. Yes	
036	Is a changing room available in the facility?	1. No<br>2. Yes	
037	Were you medically screened before employment?	1. No<br>2. Yes	
038	Are you understaffed in your department?	1. No<br>2. Yes	

SECTION V : BEHAVIOURAL FACTORS

No.	Question	Coding category	Skip
039	Are you familiar with the occupational health and safety policy?	1. Not at all<br>2. A little bit<br>3. Moderately<br>4. Quite a bit<br>5. Extremely	
040	How often do you use protocols for work procedures?	1. Never<br>2. Rarely<br>3. Sometimes<br>4. Most of the time<br>5. Always	
041	How often do you use the prescribed equipment for work?	1. Never<br>2. Rarely<br>3. Sometimes<br>4. Most of the time<br>5. Always	
042	How often do you use PPE for work procedures?	1. Never<br>2. Rarely	

		3. Sometimes<br>4. Most of the time<br>5. Always	
043	Do you perform hand hygiene during work procedures?	1. Never<br>2. Rarely<br>3. Sometimes<br>4. Most of the times<br>5. Always	
044	Do you adhere to standard precautions during work procedures?	1. Never<br>2. Rarely<br>3. Sometimes<br>4. Most of the times<br>5. Always	

SECTION VI : INTERVENTION STRATEGIES

No.	Question	Coding category	Skip
045	Have you been trained on the machinery and equipment used for your work?	1. No<br>2. Yes	
046	Have you been trained on injury management?	1. No<br>2. Yes	
047	Have been trained on proper ways of wearing PPE?	1. No<br>2. Yes	
048	Have you had training on equipment used in the transportation of patients or other heavy objects?	1. No<br>2. Yes	
049	Have you been trained on standard precautions?	1. No<br>2. Yes	
050	Have you been trained on hand washing?	1. No<br>2. Yes	

SECTION VII : NEEDLESTICK INJURIES

No.	Question	Coding category	Skip
051	Do you use or handle syringes in your line of work?	1. No<br>2. Yes	
052	In all the years of your working life, how many needlestick injuries did you experience?	1. 0<br>2. 1<br>3. 2<br>4. 3<br>5. 4<br>6. 5 and more	
053	Over the past 12 months, how many needlestick injuries did you experience while working?	1. 0<br>2. 1<br>3. 2<br>4. 3<br>5. 4<br>6. 5 and more	

054	Does your facility have a system for reporting needlestick injuries?	1. No<br>2. Yes	
055	Have you had training on the handling of wastes involving needles?	1. No<br>2. Yes	
056	Does your facility have special containers for keeping needle and sharp objects wastes?	1. No<br>2. Yes	
057	Do you dispose sharp needles in regular waste bins?	1. No<br>2. Yes	

SECTION VIII : SPLASH OF BODY FLUIDS

No.	Question	Coding category	Skip
058	Do you use or handle blood or other body fluids, or materials visibly soiled with blood, urine, faeces or vomit in your line of work?	1. No<br>2. Yes	
059	In all the years of your working life, how many times have you experience splash of blood or body fluids?	1. 0<br>2. 1<br>3. 2<br>4. 3<br>5. 4<br>6. 5 and more	
060	Over the past 12 months, how many times have you experience splash of blood or body fluids?	1. 0<br>2. 1<br>3. 2<br>4. 3<br>5. 4<br>6. 5 and more	If **answer** is **0**, skip to question (062)
061	If you did experienced splash of body fluid in the past 12 months, which fluid (s) was involved in your recent exposure?	1. Blood<br>2. Urine<br>3. Sputum<br>4. Saliva<br>5. Vomit<br>6. Amniotic fluid<br>7. Others	
062	Is a system in place for the report of splash of body fluids?	1. No<br>2. Yes	

SECTION IX : WORKPLACE VIOLENCE

No.	Question	Coding category	Skip
063	Is your facility safe for work?	1. No<br>2. Yes	
064	Do you feel adequately secured while working?	1. No<br>2. Yes	
065	Have you been trained on ways of reporting incidence such as violence at work?	1. No<br>2. Yes	
066	Is there a policy on workplace violence?	1. No<br>2. Yes	

		3. Don't know	
067	Have you been trained to recognize warning signs of violence?	1. No<br>4. Yes	
068	In your working life, how many times have you been physically attacked in your workplace?	1. 0<br>2. 1<br>3. 2<br>4. 3<br>5. 4<br>6. 5 and more	
069	In the past 12 months, how many times have been physically attacked in your workplace?	1. 0<br>2. 1<br>3. 2<br>4. 3<br>5. 4<br>6. 5 and more	If **answer** is **0**, skip to question (071)
070	Which people attacked you in your last violent incident?	1. Patient<br>2. Relative of patient<br>3. Staff member<br>4. Supervisor<br>5. General public	
077pa	After your recent exposure to physical assault, have you experienced repeated, disturbing memories, thoughts, or images of the event?	1. Not at all<br>2. A little bit<br>3. Moderately<br>4. Quite a bit<br>Extremely	
078pa	After your recent exposure to physical assault, have you experienced avoiding thinking or talking about the event or avoiding having feeling related to it?	1. Not at all<br>2. A little bit<br>3. Moderately<br>4. Quite a bit<br>Extremely	
079pa	After your recent exposure to physical assault, have you experienced being 'super alert' or watchful and on the guard?	1. Not at all<br>2. A little bit<br>3. Moderately<br>4. Quite a bit<br>Extremely	
071	In your working life, how many times have you been verbally abused in your workplace?	1. 0<br>2. 1<br>3. 2<br>4. 3<br>5. 4<br>6. 5 and more	
072	In the past 12 months, how many times have been verbally abused in your workplace?	1. 0<br>2. 1<br>3. 2<br>4. 3<br>5. 4<br>6. 5 and more	If **answer** is **0**, skip to question (074)
073	Which people verbally abused you in last incident?	1. Patient<br>2. Relative of patient<br>3. Staff member<br>4. Supervisor<br>5. General public	

077va	After your recent exposure to verbal abuse, have you experienced repeated, disturbing memories, thoughts, or images of the event?	5. Not at all<br>6. A little bit<br>7. Moderately<br>8. Quite a bit<br>Extremely	
078va	After your recent exposure to verbal abuse, have you experienced avoiding thinking or talking about the event or avoiding having feeling related to it?	5. Not at all<br>6. A little bit<br>7. Moderately<br>8. Quite a bit<br>Extremely	
079va	After your recent exposure to verbal abuse, have you experienced being 'super alert' or watchful and on the guard?	5. Not at all<br>6. A little bit<br>7. Moderately<br>8. Quite a bit<br>Extremely	
074	In your working life, how many times have you been sexually harassed in your workplace?	1. 0<br>2. 1<br>3. 2<br>4. 3<br>5. 4<br>6. 5 and more	
075	In the past 12 months, how many times have been sexually harassed in your workplace?	1. 0<br>2. 1<br>3. 2<br>4. 3<br>5. 4<br>6. 5 and more	If **answer** is **0**, skip question (080)
076	Who sexually harassed you in your last incident?	1. Patient<br>2. Relative of patient<br>3. Staff member<br>4. Supervisor<br>5. General public	
077sa	After your recent exposure to sexual harassment, have you experienced repeated, disturbing memories, thoughts, or images of the event?	9. Not at all<br>10. A little bit<br>11. Moderately<br>12. Quite a bit<br>13. Extremely	
078sa	After your recent exposure to sexual harassment, have you experienced avoiding thinking or talking about the event or avoiding having feeling related to it?	9. Not at all<br>10. A little bit<br>11. Moderately<br>12. Quite a bit<br>13. Extremely	
079sa	After your recent exposure to sexual harassment, have you experienced being 'super alert' or watchful and on the guard?	9. Not at all<br>10. A little bit<br>11. Moderately<br>12. Quite a bit<br>13. Extremely	

SECTION X : LOWER BACK PAIN

No.	Question	Coding category	Skip
080	How often does your work involve prolonged sitting?	1. Never<br>2. Rarely<br>3. Sometimes<br>4. Most of the time<br>5. Always	
081	How often does your work involve prolonged standing?	1. Never<br>2. Rarely<br>3. Sometimes<br>4. Most of the time<br>5. Always	
082	How often do you lift/reposition heavy objects?	1. Never<br>2. Rarely<br>3. Sometimes<br>4. Most of the time<br>5. Always	
083	How often does your work involve transferring patient?	1. Never<br>2. Rarely<br>3. Sometimes<br>4. Most of the time<br>5. Always	
084	How often do you bend/twist while working?	1. Never<br>2. Rarely<br>3. Sometimes<br>4. Most of the time<br>5. Always	
085	How often does your work involve pushing objects	1. Never<br>2. Rarely<br>3. Sometimes<br>4. Most of the time<br>5. Always	
086	How often does your work involve pulling objects	1. Never<br>2. Rarely<br>3. Sometimes<br>4. Most of the time<br>5. Always	
087	Does your job involves being in awkward positions?	1. No<br>2. Yes	
088	How often do you use aids to carry heavy objects including patients?	1. Never<br>2. Rarely<br>3. Sometimes<br>4. Most of the time<br>5. Always	
089	How often do you consciously maintain good posture while working?	1. Never<br>2. Rarely<br>3. Sometimes<br>4. Most of the time	

		5. Always	
090	Have you been trained on good working postures?	1. No<br>2. Yes	
091	Since you started working in health care facility, have you ever experienced lower back pain?	1. No<br>2. Yes	If **answer** is **No**, survey has ended
092	How long did you have the lower back pain?	1. Less than a month<br>2. 1-2 months<br>3. 3-4 months<br>4. 5 months and above.	
093	Did the pain begin after a work-related injury?	1. No<br>2. Yes	
094	After your last exposure to lower back pain, how often did you dream about getting another job that will better suit your personal needs?	1. Never<br>2. Rarely<br>3. Sometimes<br>4. Most of the times<br>5. Always	
095	After your last exposure to lower back pain, how often were you frustrated when not given the opportunity at work to achieve your personal work-related goals?	1. Never<br>2. Rarely<br>3. Sometimes<br>4. Most of the times<br>5. Always	
096	After your last exposure to lower back pain, how often did you considered leaving your job?	1. Never<br>2. Rarely<br>3. Sometimes<br>4. Most of the times<br>5. Always	
097	After your last exposure to lower back pain, how likely were you to accept another job at the same compensation level should it be offered to you?	1. Never<br>2. Rarely<br>3. Sometimes<br>4. Most of the times<br>5. Always	
098	After your last exposure to lower back pain, how often did you look forward to another day at work?	1. Never<br>2. Rarely<br>3. Sometimes<br>4. Most of the times<br>5. Always	
099	To what extend is your current job satisfying your personal needs?	1. Never<br>2. Rarely<br>3. Sometimes<br>4. Most of the times<br>5. Always	

THANK YOU FOR PARTICIPATING IN THIS RESEARCH
